# Attitudes towards euthanasia in severely ill and dementia patients and cremation in Cyprus: a population-based survey

**DOI:** 10.1186/1471-2458-13-878

**Published:** 2013-09-23

**Authors:** Anastasios Televantos, Michael A Talias, Marianna Charalambous, Elpidoforos S Soteriades

**Affiliations:** 1 Cyprus Institute of Biomedical Sciences (CIBS), Athienitis Strovolos Park, 2A Elia Venezi Street, 2nd Fl. Office 206, 2042, Strovolos, Nicosia, Cyprus; 2 Open University of Cyprus, Healthcare Management Program, 2252, Latsia, Nicosia, Cyprus; 3 Harvard School of Public Health, Department of Environmental Health, Environmental and Occupational Medicine and Epidemiology (EOME), Boston, MA, USA

**Keywords:** Euthanasia, Cremation, Religion, Population survey, Cyprus

## Abstract

**Background:**

Population studies on end-of-life decisions have not been conducted in Cyprus. Our study aim was to evaluate the beliefs and attitudes of Greek Cypriots towards end-of-life issues regarding euthanasia and cremation.

**Methods:**

A population-based telephone survey was conducted in Cyprus. One thousand randomly selected individuals from the population of Cyprus age 20 years or older were invited to participate. Beliefs and attitudes on end-of-life decisions were collected using an anonymous and validated questionnaire. Statistical analyses included cross-tabulations, Pearson’s chi-square tests and multivariable-adjusted logistic regression models.

**Results:**

A total of 308 males and 689 females participated in the survey. About 70% of the respondents did not support euthanasia for people with incurable illness and/or elders with dementia when requested by them and 77% did not support euthanasia for people with incurable illness and/or elders with dementia when requested by relatives. Regarding cremation, 78% were against and only 14% reported being in favor. Further statistical analyses showed that male gender, being single and having reached higher educational level were factors positively associated with support for euthanasia in a statistically significant fashion. On the contrary, the more religiosity expressed by study participants, the less support they reported for euthanasia or cremation.

**Conclusions:**

The vast majority of Greek Cypriots does not support euthanasia for people with incurable illness and/or elders with dementia and also do not support cremation. Certain demographic characteristics such as age and education have a positive influence towards attitudes for euthanasia and cremation, while religiosity exerts a strong negative influence on the above. Family bonding as well as social and cultural traditions may also play a role although not comprehensively evaluated in the current study.

## Background

The impressive scientific advances in genetics and medical technology have contributed to enhanced enthusiasm among the general public for the human potential and a great optimism for improving the health and quality of life in our societies [1]. However, many of these advances are coupled with new ethical challenges leading to heated discussions among scientists and the public often involving matters of religious faith and end-of-life decisions. Difficult ethical dilemmas inevitably accompany the unlimited growth of technology especially in the field of medicine [2].

The first proposed use of anesthetics to end the lives of patients with painful and incurable disease dates back to 1870, initiating extensive debates about euthanasia [3]. Definitions on euthanasia vary significantly ranging from simple assisted suicide to physician-assisted suicide, while many define euthanasia as the painless killing of a patient suffering from an incurable and painful illness or being in an irreversible coma. Studies on euthanasia report strong differences in attitudes among medical practitioners [4-6] and also reveal general disagreement on the morality of euthanasia among different societies [7,8]. For example, a study conducted in the US revealed that many professionals find it difficult to assist in euthanasia and regretted their decision to be involved [9], while another recent study showed that the majority of Greek physicians do not agree with euthanasia [10].

In the Netherlands euthanasia has been socially accepted and openly practiced for many years and as of 2011 it is also legalized [11]. Other studies reveal that attitudes towards euthanasia are heavily influenced by religion, with religious people mainly opposing euthanasia [12-14].

On the contrary, cremation has been widely practiced even among ancient civilizations. Nevertheless, in countries with Christian culture, cremation has historically been discouraged. In other religions, such as Hinduism and Buddhism, cremation was mandated [15]. During the past few decades we have witnessed a rapid increase of acceptance for cremation around the world including the USA and many parts of Europe [16]. Factors such as cost and land conservation are the main reasons influencing support for cremation, while religious beliefs constitute one of the main barriers of acceptance [17].

Despite the above, end-of-life decisions have not been the center of public debate in Cyprus until quite recently, when the Cyprus National Bioethics Committee was established in 2001. The terms euthanasia and cremation are not used in the Cyprus legislation and there are no laws that permit euthanasia or cremation [18]. Professional codes of practice also do not allow euthanasia or cremation. What is more, the Orthodox Church in Cyprus does not recognize cremation as a religiously acceptable practice. People in Cyprus tend to view euthanasia as an unjustified suicide attempt and many believe that those who help people end their lives are more or less participating in a case of murder. Furthermore, strong societal beliefs are supporting the idea that euthanasia attempts reflect lost of religious faith. In addition, cremation is denounced not only by the Orthodox church but also by strong beliefs embedded in the Greek culture mandating high respect for the deceased body mostly expressed during the burial services [see also Greek Mythology: Antigone (Sofokles)].

Population studies on beliefs and attitudes regarding euthanasia and cremation have not been previously reported in Cyprus. The objective of our study was to examine the beliefs and attitudes of the Cyprus population with respect to euthanasia and cremation and explore their potential association with other population characteristics including, age, education and religiousness.

## Methods

The study was conducted in the Republic of Cyprus through a telephone survey using a validated anonymous questionnaire during the period of April 2007 to May 2007.

### Study sample

The sample consisted of one thousand people (n = 1000) over the age of twenty living in Cyprus. The sample was selected based on the population ratio of each district. More specifically, the sample consisted of four hundred respondents from the city and district of Nicosia (the capital city), three hundred respondents from the city and district of Limassol, two hundred from Larnaca and Famagusta, and one hundred respondents from the city and district of Paphos, respectively. The respondents were selected at random one from each page of the phone directory and survey questionnaires were completed throughout the day over the phone. In order to collect 1.000 completed questionnaires, a total of 2.027 phone calls were placed leading to a response rate of 49%. All phone calls were completed by a single field researcher.

### Questionnaire – data collection

The questionnaire consisted of a total of 14 questions (Additional file 1). Six questions referred to demographic characteristics of the respondents and 8 questions considered bioethical questions including end-of-life decisions about euthanasia and cremation. Responses to each close-ended question were given on a 4-point scale. However, we did not provide a specific definition of euthanasia to the survey respondents before asking them to respond to the following question: “Are you in favor of euthanasia for people with incurable illness or elders with dementia if requested by themselves” and “if requested by their relative”. Using the following approach, we relied on the respondents’ understanding of the broad term “euthanasia” in order to provide their beliefs and attitudes on the above issue. Responses were documented on paper and then entered into a computerized electronic database.

### Statistical analyses

The recorded data were analyzed using the open source “R” programming language. A contingency table analysis was performed to examine the association of population demographics with beliefs and attitudes on euthanasia and cremation. The joint frequency distribution was analyzed using Pearson’s chi-square test to determine whether the variables were statistically independent or whether they were associated. The statistical significant level was set at p = 0.05 and was two sided for all tests.

## Results

A total of 1.000 individuals completed the survey over the phone (308 males, 689 females). The demographic characteristics of the study population are presented in Table 1.

**Table 1:** Demographic characteristics of the study population

**Characteristics**	**Number**	**Percentage (%)**
**Age categories**		
20 – 40 years old	275	27.5
40 – 60 years old	436	43.7
> 60 years old	287	28.7
**Gender**		
Male	308	31.0
Female	689	69.0
**Marital status**		
Single – other	94	9.4
Married	905	90.6
**Education**		
Elementary school	202	20.3
High school	474	47.6
University	320	32.1
**Occupation**		
Medical/Paramedical	46	4.6
Other	951	95.4
**Reported Religiousness**		
Much	580	58.3
Little	360	36.2
None	55	5.5

In Table 2 we present the association between basic demographics and population beliefs and attitudes about their support for euthanasia. Whenever euthanasia is mentioned in the results section and/or throughout the manuscript, we refer to a combined term of euthanasia for people with incurable illness and/or elders with dementia. In Table 2 we document participants’ attitudes on euthanasia for people with incurable illness and/or elders with dementia when requested by them. In general, the higher the educational level of the respondents the more support they expressed towards euthanasia. Males and those who were not married reported statistically significantly higher support for euthanasia. On the contrary, the more religiousness reported by the respondents the less favorable support they expressed for euthanasia. Age also appeared to be a factor affecting beliefs and attitudes on euthanasia. It appeared that younger respondents (< 40) were more in favor towards euthanasia as compared with older respondents however the difference was not statistically significant. Overall, 70% of study participants, regardless of their particular characteristics, reported no support for euthanasia.

**Table 2:** Association of population demographics with attitudes on euthanasia

**Demographics**	**Much support n (%)**	**Little support n (%)**	**No support n (%)**	**Do not know/answer n (%)**	**p - value**
**Total**	191 (19.1)	38 (3.8)	700 (70.1)	69 (6.9)	-
**Age categories**					
20 – 40 years old	65 (23.6)	8 (2.9)	181 (65.8)	21 (7.6)	
40 – 60 years old	80 (18.3)	20 (4.6)	302 (69.3)	34 (7.8)	
> 60 years old	46 (16.0)	10 (3.5)	217 (75.6)	14 (4.9)	0.11
**Gender**					
Male	90 (29.2)	15 (4.9)	183 (59.4)	20 (6.5)	
Female	101 (14.7)	23 (3.3)	516 (74.9)	49 (7.1)	< 0.0001
**Education**					
Elementary school	27 (13.4)	4 (2.0)	161 (79.7)	10 (4.9)	
High School	81 (17.1)	17 (3.6)	345 (72.8)	31 (6.5)	
University	82 (25.6)	17 (5.3)	193 (60.3)	28 (8.7)	< 0.0001
**Marital status**					
Married	172 (19.0)	37 (4.1)	641 (70.8)	55 (6.1)	
Single – Other	20 (21.2)	1(1.1)	59 (62.8)	14 (14.9)	0.01
**Occupation**					
Medical/Paramedical	9 (19.6)	1 (2.2)	34 (73.9)	2 (4.3)	
Other	182 (19.1)	37 (3.9)	665 (69.9)	67 (7.0)	0.94
**Reported Religiousness**					
Much	77 (13.3)	11 (1.9)	457 (78.8)	35 (6.0)	
Little	91 (25.3)	23 (6.4)	217 (60.3)	29 (8.0)	
None	22 (39.7)	4 (6.9)	24 (43.6)	5 (9.1)	< 0.0001

Table columns represent responses to the question “Do you support euthanasia for people with incurable illness and/or elders with dementia when requested by them?”.

In Table 3 we present the association between demographics and population beliefs and attitudes about the study participants’ support for euthanasia for people with incurable illness and/or elders with dementia when requested by their relatives. Overall, 77% of the population expressed no support for euthanasia when requested by relatives. Statistically significant differences were identical with those detected in Table 2, referring to euthanasia requested by people themselves as opposed to the request originating from their relatives.

**Table 3:** Association of population demographics with attitudes on euthanasia

**Demographics**	**Much support n (%)**	**Little support n (%)**	**No support n (%)**	**Do not know/answer n (%)**	**p - value**
**Total**	98 (9.8)	43 (4.3)	769 (77.0)	88 (8.8)	-
**Age categories**					
20 – 40 years old	27 (9.8)	15 (5.5)	206 (74.9)	27 (9.8)	
40 – 60 years old	48 (11.0)	17 (3.9)	331 (75.9)	40 (9.2)	
> 60 years old	23 (8.0)	11 (3.8)	232 (80.8)	21 (7.3)	0.59
**Gender**					
Male	49 (15.9)	20 (6.5)	211 (68.5)	28 (9.1)	
Female	49 (7.1)	23 (3.3)	557 (80.9)	60 (8.7)	< 0.0001
**Education**					
Elementary school	18 (8.9)	2 (1.0)	169 (83.7)	13 (6.4)	
High School	41 (8.6)	21 (4.4)	371 (78.3)	41 (8.6)	< 0.0018
University	38 (11.9)	20 (6.2)	228 (71.3)	34 (10.6)	
**Marital status**					
Married	92 (10.2)	40 (4.4)	701 (77.5)	71 (7.9)	
Single – Other	6 (6.4)	3 (3.2)	68 (72.3)	17 (18.1)	< 0.02
**Occupation**					
Medical/Paramedical	2 (4.3)	1 (2.2)	40 (87.0)	3 (6.5)	
Other	96 (10.1)	42 (4.4)	728 (76.6)	85 (8.9)	0.55
**Reported Religiousness**					
Much	36 (6.2)	15 (2.6)	486 (83.8)	43 (7.4)	
Little	50 (13.9)	23 (6.4)	250 (69.4)	37 (10.3)	
None	12 (20.7)	5 (8.6)	33 (56.9)	8 (13.8)	< 0.0001

Table columns represent responses to the question “Do you support euthanasia for people with incurable illness and/or elders with dementia when requested by relatives?”.

Population attitudes towards cremation are presented in Table 4. The results reveal that the majority of Cypriots (78%) are not in support for cremation. As religiousness increases, the support for cremation decreases in a statistically significant fashion. On the contrary, the higher the educational level of the respondent, male gender and being single, were factors contributing to statistically significantly favorable attitudes for cremation. In general, findings on cremation were similar to those documented for euthanasia in Tables 2 and 3.

**Table 4:** Association of population demographics with attitudes on cremation

**Demographics**	**Much support n (%)**	**Little support n (%)**	**No support n (%)**	**Do not know/answer n (%)**	**p - value**
**Total**	78 (7.8)	66 (6.6)	781 (78.2)	73 (7.3)	-
**Age categories**					
20 – 40 years old	21 (7.6)	25 (9.1)	209 (76.0)	20 (7.3)	
40 – 60 years old	31 (7.1)	25 (5.7)	347 (79.6)	33 (7.6)	
> 60 years old	26 (9.1)	16 (5.6)	225 (78.4)	20 (6.9)	0.57
**Gender**					
Male	38 (12.3)	32 (10.4)	208 (67.5)	30 (9.7)	
Female	40 (5.8)	34 (4.9)	572 (83.0)	43 (6.2)	< 0.0001
**Education**					
Elementary school	11 (5.4)	9 (4.5)	173 (85.6)	9 (4.4)	
High School	40 (8.4)	21 (4.4)	379 (80.0)	34 (7.2)	
University	27 (8.5)	35 (10.9)	228 (71.2)	30 (9.4)	< 0.0006
**Marital status**					
Married	74 (8.1)	52 (6.5)	711 (78.6)	68 (7.5)	
Single – Other	4 (4.3)	14 (14.9)	70 (74.5)	6 (6.4)	0.012
**Occupation**					
Medical/Paramedical	3 (6.5)	2 (4.3)	37 (80.4)	4 (8.7)	
Other	75 (7.9)	64 (6.7)	743 (78.1)	69 (7.2)	0.93
**Reported Religiousness**					
Much	25 (4.3)	34 (5.9)	496 (85.5)	25 (4.3)	
Little	39 (10.8)	28 (7.8)	253 (70.3)	40 (11.1)	
None	14 (24.1)	4 (6.9)	30 (54.5)	7 (12.7)	< 0.0001

Table columns represent responses to the question “Do you support cremation instead of burial?”.

In Table 5 we present the results of multivariable-adjusted logistic regression models examining the association between basic demographics and attitudes for euthanasia and cremation. We found that females were 2.5 times more likely to be against euthanasia or cremation compared to men. Also, elderly study participants (older than 60 years of age) had a 50% higher likelihood of being against euthanasia compared to men. On the contrary, study participants who had higher education (e.g. university level education) were 2 – 2.5 times more likely to support euthanasia or cremation compared to study participants who had completed only elementary school. In addition, those who reported not being religious, were about 4 to 5 times more likely to support euthanasia and cremation compared to those who reported being religious at a high level. In categorical level variables such as education and religiousness, we also noted a dose–response relationship between the different levels and the corresponding support for euthanasia and cremation.

**Table 5:** Odds ratios and 95% confidence intervals from multivariable logistic regression models examining the association between population characteristics and support for euthanasia and cremation in Cyprus

**Population characteristics**	**Against euthanasia requested**	**Against euthanasia requested**	**Against cremation**
	**by people themselves**	**for people by relatives**	**odds ratio (95% CI)**
	**odds ratio (95% CI)**	**odds ratio (95% CI)**	
**Age categories**			
20 – 40 years old (reference)	–	–	–
40 – 60 years old	1.21 (0.86 – 1.72)	1.03 (0.67 – 1.56)	1.36 (0.89 – 2.08)
> 60 years old	1.49 (1.01 – 2.22)	1.34 (0.83 – 2.19)	1.17 (0.74 – 1.85)
**Gender**			
Male (reference)	–	–	–
Female	2.36 (1.74 – 3.20)	2.47 (1.72 – 3.56)	2.44 (1.70 – 3.50)
**Education**			
Elementary school (reference)	–	–	–
High School	0.69 (0.44 – 1.07)	0.73 (0.42 – 1.22)	0.74 (0.43 – 1.25)
University	0.40 (0.25 – 0.63)	0.50 (0.28 – 0.84)	0.46 (0.26 – 0.77)
**Marital status**			
Married (reference)	–	–	–
Single	1.04 (0.64 – 1.78)	1.61 (0.83 – 3.52)	0.68 (0.40 – 1.21)
**Reported Religiousness**			
Much (reference)	–	–	–
Little	0.39 (0.28 – 0.53)	0.38 (0.26 – 0.56)	0.49 (0.34 – 0.72)
None	0.21 (0.12 – 0.36)	0.23 (0.12 – 0.45)	0.25 (0.14 – 0.48)

## Discussion

To our knowledge this is the first study in Cyprus examining population beliefs and attitudes with respect to end-of-life decisions about euthanasia and cremation. Our survey showed that Greek Cypriots are opposed to euthanasia for themselves even if they are terminally ill or suffer from dementia and they are also opposed to euthanasia for their close relatives who have similar conditions. In addition Greek Cypriots, in their vast majority, are opposed to cremation.

In particular, our study reveals that only 23% of the study respondents favored euthanasia. This finding is in stark contrast to the general public attitudes observed in other European countries where the percentages in favor for euthanasia are much higher, among which the highest in countries such as Belgium (72%) and the Netherlands (80%) [19]. Studies also reveal a marked increase over the last few years on the acceptance of euthanasia in most European countries. Weak religious belief is found to be the most important factor associated with this increase [20,21]. Nevertheless, comparisons of our results with other European studies are mainly restricted by the fact that our study questionnaire combined euthanasia for people with incurable illness and elders with dementia. Deep religious beliefs, social/cultural traditions and strong family bonds among Greek Cypriots may be the most important contributing factors affecting Cyprus population attitudes and beliefs against euthanasia observed in our study.

The percentage in favor of euthanasia when requested by a family member decreases to 14% (Table 3) suggesting again that certain population characteristics including strong family traditions prevail among Greek Cypriots and strongly influence their attitudes on euthanasia. Similarly to the above, beliefs and attitudes on euthanasia among family members in other European countries, are much higher than those observed in our study [22-24]. However, it is notable that young age and higher educational levels among our study respondents were significantly associated with higher support for euthanasia, suggesting that the new and more educated generation of Cypriot society is distancing itself from traditional and cultural beliefs that influence the above opposition.

With respect to cremation, only about 14% of the Greek Cypriots are in favor, one of the lowest percentages in the European Union. Cremation in most European countries has come to appeal to the majority of the population. In particular the percentages in favor are much higher in countries such as the United Kingdom (71%), the Netherlands (50%) and Switzerland (76%) [16,25].

Certain limitations of our study should be acknowledged. Since this is the first survey on such issues in Cyprus, we are unable to perform any comparisons over time. Furthermore, our study population in Cyprus was relatively homogeneous with respect to religiousness and cultural background, thereby limiting our capacity to perform comparisons between different denominations or cultural groups. Therefore, further studies may be needed to conduct subgroups analyses. What is more, the degree of religiousness was subjectively measured with only one question and therefore we did not explore this important population characteristic in depth. Our survey included a “double” question on euthanasia since we asked participants whether they would be in favor of euthanasia for people with incurable illness and/or elders with dementia at the same time. As such, we were unable to unravel the attitudes of study participants on euthanasia for those two separate groups. Our survey was based on a telephone sampling methodology among households having a home telephone line, an approach which might have excluded those who have only cellular phones. In addition, the overall response rate of our telephone survey was not very high although at an acceptable level (reached 49%). Finally, we failed to include in our questionnaire inquiries about the issue of palliative sedation.

## Conclusions

In conclusion, in our study, we provide population-based estimates on the beliefs and attitudes of Greek Cypriots with respect to euthanasia and cremation in association with certain population characteristics including age, education and religiosity. The vast majority of Greek Cypriots are against euthanasia and cremation and their beliefs and attitudes appear to be strongly influenced by the degree of religiosity expressed in our survey. In addition, family values appear to play an important role in the above opposition since relatives were strongly opposed to euthanasia for their loved ones. Based on the above, we also believe that other societal factors including long held social and cultural norms (high respect for those deceased, beliefs about the holiness of body) may also play an important role on the above findings. On the contrary, young age and higher educational level were factors positively influencing population attitudes towards euthanasia and cremation. The fact that we requested study participants to respond to our questions without providing specific definitions about euthanasia and cremation, warrants further exploratory/analytical research to clarify the origin of such strong beliefs and compare them with other societies. Finally, our study findings reveal significant differences between population views on the above issues between Cyprus and other European countries.

## Competing interests

The authors declare that they have no competing interests.

## Authors’ contributions

AT conceived of the idea for the study. AT and ESS supervised the data collection and management process. MAT and MC performed the statistical analyses. All authors reviewed and interpreted the statistical analyses. AT and ESS wrote the first draft of the manuscript. All authors contributed to the final version of the manuscript. All authors read and approved the final version of the manuscript.

## Pre-publication history

The pre-publication history for this paper can be accessed here:

http://www.biomedcentral.com/1471-2458/13/878/prepub

## Supplementary Material

Additional file 1 Questionnaire on bioethical issues. Click here for file
