# Supplementary material for: Attitudes towards euthanasia in severely ill and dementia patients and cremation in Cyprus: a population-based survey
Source: BMC Public Health. 2013 Sep 23;13:878. doi: 10.1186/1471-2458-13-878 (PMC3852336; doi:10.1186/1471-2458-13-878)
Supplement: Additional file 1 — Questionnaire on bioethical issues. [file 1471-2458-13-878-S1.doc]

**Additional File 1:**

**Questionnaire on Bioethical Issues**

**________________________________________________**

Demographics

1. Gender Male □ Female □

2. Age group 20 - 40 □ 40 -60 □ 60 + □

3. Educational level Elementary level □ High School □ University □

4. Religiousness Very □ Somewhat □ Not at all □

5. Occupation Medical / Paramedics □ Other □

6. Marital status Married □ Single □

________________________________________________________________________

Questions

7. Would you accept to offer your organs or your beloved persons’ organs when a clinical death occurs?

Highly in favor □ Low in favor □ Not at all □ Do not know □

8. Would you or your loved persons accept to get organ transplants, if there was no other way of treating a serious illness?

Highly in favor □ Low in favor □ Not at all □ Do not know □

9. Are you in favor of cremation instead of burial?

Highly in favor □ Low in favor □ Not at all □ Do not know □

10. Are you in favor of euthanasia for people with incurable illness or elders with dementia if:

a) It is requested by themselves:

Highly in favor □ Low in favor □ Not at all □ Do not know □

b) It is requested by their relatives:

Highly in favor □ Low in favor □ Not at all □ Do not know □

11. In the case that a fetus has been diagnosed with a serious health problem, would you give your consent for an abortion?

Highly in favor □ Low in favor □ Not at all □ Do not know □

12. If you had no children would you accept to do In Vitro Fertilization (IVF) if:

a) You know that many embryos will be lost until you succeed:

Highly in favor □ Low in favor □ Not at all □ Do not know □

b) It is needed to use foreign sperm or foreign woman’s egg:

Highly in favor □ Low in favor □ Not at all □ Do not know □
